# Gluten-Free Diet Alters the Gut Microbiome in Women with Autoimmune Thyroiditis

**DOI:** 10.3390/nu16050685

**Published:** 2024-02-28

**Authors:** Aleksandra Rodziewicz, Adrian Szewczyk, Ewa Bryl

**Affiliations:** 1 Department of Pathology and Experimental Rheumatology, Faculty of Medicine, Medical University of Gdańsk, 80-210 Gdańsk, Poland; aleksandra.rodziewicz@gumed.edu.pl; 2 Department of Pathophysiology, Faculty of Medicine, Medical University of Gdańsk, 80-210 Gdańsk, Poland; 3 Department of Physical Chemistry, Faculty of Pharmacy, Medical University of Gdańsk, 80-416 Gdańsk, Poland; adrian.szewczyk@gumed.edu.pl

**Keywords:** diet, gut microbiome, autoimmune thyroiditis, gluten-free diet

## Abstract

The gut microbiome may contribute to the development of autoimmune diseases, such as autoimmune thyroiditis (AIT). Diet has a critical impact on the gut microbiome, and it has been shown that a gluten-free diet can negatively affect its composition. A gluten-free diet is popular among patients, and therefore the aim of this study was to check whether it affects thyroid function and gut microbiome composition in AIT. Thirty-one women with AIT complied with a gluten-free diet for 8 weeks. After the first 4 weeks, participants were divided into two groups: the first group received gluten in capsules and the other one—rice starch (placebo). Blood and stool samples were examined before diet (T_0_), after 4 weeks (T_1_) and after 8 weeks of diet (T_2_). The only significant difference in blood parameters was observed between T_1_ and T_2_ in the placebo group for the thyroid peroxidase antibody level. After the first 4 weeks, a significant increase in *Desulfobacterota*, *Proteobacteria*, *Prevotella* and *Parasutterella* and a significant decrease in *Actinobacteriota*, *Coriobacteriaceae* and *Bifidobacterium* were observed. The detected microbiome alterations may indicate increasing inflammation; however, further research is required, and for now, a gluten-free diet should be used cautiously in AIT.

## 1. Introduction

Autoimmune thyroiditis (AIT), also known as Hashimoto’s thyroiditis (HT), is an organ-specific autoimmune disease. It is estimated that autoimmune thyroid diseases affect 1.5% of the population, mainly women [1,2,3]. It is also estimated that the incidence of autoimmune hypothyroidism is 350/100,000/year in women and 80/100,000/year in men [1]. In regions rich in iodine, AIT is the most common cause of hypothyroidism [4]. As with most autoimmune diseases, the pathogenesis is unclear. The influence of many factors is emphasized, including genetic and environmental factors, such as selenium deficiency, iodine excess, stress and infections.

Gut microbiome disorders may be one of these factors. There is growing evidence that the gut microbiome interacts with the human immune system and may contribute to the development of autoimmune diseases [5]. Intestinal dysbiosis has also been detected in AIT patients [6].

Diet has a direct effect on the gut microbiome and thus on general health. The gut microbiome composition depends on the fiber supply in vegetables, fruits and whole grains, as well as the amount of simple sugars and the type and amount of dietary fat [7,8].

A gluten-free diet is becoming more and more popular. Patients eliminate gluten on their own, hoping to reduce inflammation and improve their well-being. Presently, only two studies have investigated the influence of a gluten-free diet in AIT in patients without gluten-related conditions [9,10]; however, the obtained results are inconsistent. In the first study, Krysiak and Szkróbka reported that 6 months of diet decreased thyroid antibody levels: thyroid peroxidase antibodies (TPOAb or anti-TPO antibodies) and thyroglobulin antibodies (TgAb or anti-Tg antibodies) [9]. In another study, conducted by Pobłocki et. al., a significant decrease in thyroid-stimulating hormone (TSH) after 12 months of gluten-free diet was demonstrated.

Moreover, a relationship between AIT and celiac disease has been demonstrated. The prevalence of celiac disease is higher among people with AIT compared to the general population. According to various studies, it ranges from 2% to 5% among children, up to 7%, while in the general population it is 1% [11]. This may explain the improvement in health and well-being after eliminating gluten in some AIT patients. Some researchers even suggest that all AIT patients should be tested for celiac disease [12]. Another explanation for improvement in a gluten-free diet may be non-celiac gluten sensitivity (NCGS) [13]. Nevertheless, the prevalence of NCGS has not been precisely determined, primarily due to diagnostic difficulties.

Previous studies on a gluten-free diet in both healthy people and patients with celiac disease have shown that it can negatively affect the gut microbiome composition after 4 weeks of intervention [14,15]. Currently, the exact impact of dietary gluten elimination on AIT patients, especially on their gut microbiome composition and blood parameters, is not known. However, the consequences of eliminating gluten for AIT patients presumably can also be unfavorable.

Therefore, the aim of this randomized double-blind intervention study was to examine whether a gluten-free diet is beneficial for AIT patients, especially regarding microbiome composition and thyroid function, and whether it can be recommended in this disease.

## 2. Materials and Methods

### 2.1. Study Participants

In this study, 31 women were enrolled in Pomeranian Voivodeship, Poland, aged between 20 and 50 years, with AIT diagnosed by an endocrinologist based on increased anti-TPO and/or anti-Tg antibody levels. To be admitted to the study, they were required to have stabilized thyroid function, i.e., thyroid hormones within a normal range and BMI indicating correct body mass, i.e., within 18.5–24.9 kg/m^2^. Their weight was checked at the beginning and at the end of the study to be sure that it had no impact on the results. All of the participants maintained a similar body mass throughout the study—1.54 kg and 5 kg were the mean and the biggest weight loss, respectively.

Exclusion criteria were newly diagnosed AIT and unregulated thyroid hormones (hypothyroidism or hyperthyroidism), concomitant celiac disease and/or wheat allergy, gluten-free diet followed during 6 months prior to enrollment, intestinal disorders, and antibiotic or probiotic therapy during 6 months prior to enrollment. Eventually, three of the initially recruited patients were not included in the study.

**Figure 1:**
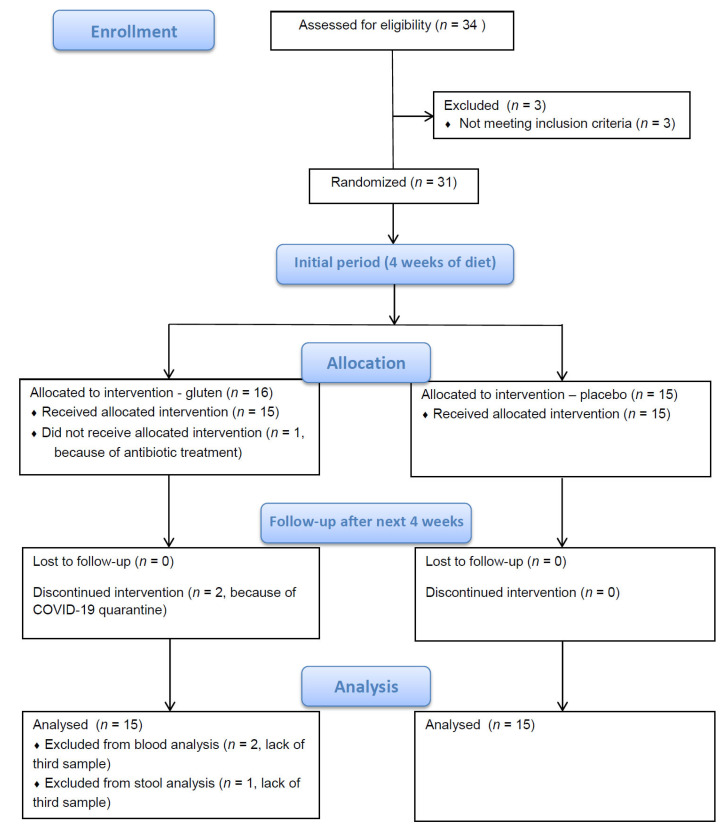
Study flow diagram.

Twenty-nine enrolled patients had been taking levothyroxine (Euthyrox or Letrox brand names), and two patients had not. One patient who received antibiotic treatment at the end of the fourth week of the study was excluded from further participation. At the end of the eighth week, 2 patients were subjected to quarantine because of COVID-19 and could not undergo the last blood sample collection. One of those patients managed to provide the last stool sample. In total, 28 patients completed the study and provided all blood and stool samples. Additionally, 1 patient provided all stool samples but not the third (last) blood sample (Figure 1).

### 2.2. Study Design

All participants were instructed to follow a normocaloric gluten-free diet for 8 weeks. Patients received comprehensive information about the gluten-free diet, additional materials about products containing gluten, and exemplary gluten-free diet plans with individually calculated caloric values.

After the first 4 weeks, participants were randomly assigned to one of two groups. Participants in both groups were of comparable age: 36.6 (7.3) and 34.6 (6.3) in the placebo and gluten groups, respectively.

Over the next 4 weeks, in addition to a gluten-free diet, one group received gluten in gastrosoluble capsules, and the second group—rice starch (placebo). This part of the study was double-blinded in order to avoid any bias of participants or the researcher. Rice starch was chosen as the placebo because it is the most easily digestible of the complex carbohydrates and thus less fermentable in the intestinal tract. The daily amount of gluten delivered in 3 capsules was 2 g, which is an equivalent of ~1 slice of white bread. This part of the intervention was based on the Di Sabatino study [16], where participants were given 4.375 g/day gluten. Two grams of gluten/day is a small amount; however, it has already been used in research, and it was chosen in this study for practical reasons and patient adherence.

The investigator met participants every 4 weeks for blood sampling (in time points T_0_, T_1_, T_2_), and during the meetings, they were asked if they had followed the diet and taken the capsules. Participants were instructed to immediately report any deviation and consumption of a product containing gluten. Participants were also asked to bring the remaining capsules if they missed a dose, which did not occur in this study.

**Figure 2:**
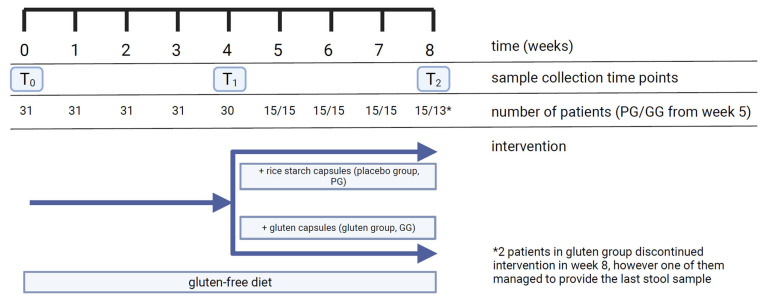
Timeline of study, including number of participants and collected samples. PG, placebo group; GG, gluten group.

Blood and stool samples were collected before diet (T_0_), after 4 weeks (T_1_) and after 8 weeks of diet (T_2_) (total of 3 samples per participant) (Figure 2).

### 2.3. Compounding of Capsules and Their Quality Assessment

Capsule compounding was performed on a manual capsule-filling machine (EPRUS 022CAPS00, Bielsko-Biała, Poland) using hydroxypropyl methylcellulose capsules size 00 (volume of 0.95 mL, NatVita, Mirków, Poland). First, the gluten powder (Amylon, Pribyslav, Czech Republic) was homogenized with a mortar for 5 min. Next, a graduated cylinder was filled with the powder to a final volume of 95 mL. The whole measured powder was then transferred onto a capsule filling panel, punched to the body of the capsules using a scraper and gently sealed with a cap. Batches of 100 capsules were obtained at once. Finally, the obtained capsules were placed in polypropylene boxes with a moisture absorber and stored in a dry, dark place before further use. The compounded capsules with rice starch (SANO GLUTEN FREE, Lipowa, Poland) were obtained in the same manner as a placebo.

The uniformity of the gluten-containing capsules was verified in accordance with European Pharmacopoeia 11.0 following monography no. 2.9.5. Uniformity of mass of single-dose preparations. Briefly, 20 randomly taken capsules were weighed (Radwag WAA 100/C/1), then opened and emptied completely. The mass of the content was expressed as the difference between the mass of a gluten-containing capsule and an empty one. The procedure was repeated eight times. The average dose of gluten was 657 ± 17 mg per capsule. None of the masses deviated from the average mass by more than the 7.5% established deviation limit, proving the uniformity of the gluten doses.

### 2.4. Laboratory Tests

Blood collection and all hormonal and metabolic tests were conducted in one of the “Diagnostyka” laboratories (Gdynia, Poland). In each blood sample, the parameters listed below were measured: erythrocyte sedimentation rate (ESR), C-reactive protein (CRP), fasting glucose, TSH, thyroid hormones thyroxine (FT4), triiodothyronine (FT3), anti-TPO antibodies and anti-Tg antibodies. Additionally, before starting the gluten-free diet, each patient was tested for celiac disease by measuring IgA anti-endomysial antibodies (EmA), IgA anti-tissue transglutaminase antibodies (tTG) and total IgA. IgG anti-gliadin antibodies (AGA) were also measured, as they are supposed to be elevated in NCGS. None of the patients had elevated levels of antibodies indicating celiac disease. One patient had elevated AGA; however, her response to the intervention did not differ significantly from that of other patients.

### 2.5. Microbiome Testing and Bioinformatic Analysis

Stool collection kits were provided by Genomed S.A. (Warsaw, Poland). Fecal samples were collected in tubes prefilled with proprietary stabilizing buffer and sent to the Genomed laboratory within 2 days after collection. Then, bacterial DNA was extracted from stool samples and stored at −80 °C.

16S rRNA sequencing was conducted after collecting all samples. Metagenomic analysis of bacterial and archaeal populations was performed based on the hypervariable V3–V4 region of the 16S rRNA gene. Specific primer sequences 341F and 785R were used to amplify the selected region and prepare the library (16S analysis). PCR was performed using Q5 Hot Start High-Fidelity 2X Master Mix (New England Biolabs, Ipswich, MA, USA) reaction conditions according to the manufacturer’s recommendations. Sequencing was performed on a MiSeq device, using paired-end (PE) technology, 2 × 300 nt, using the v3 Illumina (Illumina, San Diego, CA, USA) kit.

Bioinformatics analysis, ensuring the classification of reads to the species level, was performed using the QIIME 2 (version 2017.6.0) software package based on the Silva 138 reference sequence database. The DADA2 package was also used, which allowed for the specification of sequences of biological origin from those newly created in the sequencing process. This package was also used to extract unique sequences of biological origin, i.e., amplicon sequence variant (ASV) sequences.

### 2.6. Statistical Analysis

Analyses were conducted using the R Statistical language (version 4.1.1; R Core Team, 2021). The significance level of the statistical tests was set at α = 0.05. The normality of the distributions was tested using the Shapiro–Wilk test. Distribution measures of central tendency for numerical variables were expressed in terms of Mdn (Q1–Q3) for non-normally distributed and *M* (*SD*) for normally distributed variables, where *Mdn* is median, *Q1* is the first quartile (25%), *Q3* is the third quartile (75%), *M* is the mean value and *SD* is the standard deviation.

In the case of two independent groups with non-normally distributed variables, the Mann–Whitney *U* two-sample rank-sum test was used to compare means. For normally distributed variables, the differences between groups were estimated using the t-Welch test. Estimation of mean differences within non-normally distributed groups for two repeated measures was performed using the Wilcoxon two-sample signed-rank test. For normally distributed variables, differences between groups were estimated using the paired t-Welch test. 

In non-parametric tests, the effect size measure was estimated using *r*. The effect size *r* was calculated as the *Z* statistic divided by the square root of the sample size *N* (Equation (1)):

(1) $$r=\frac{Z}{\sqrt{N}}$$

The *Z* value was extracted from the *wilcoxsign_test*() function of {coin} package. The *r* value varies from 0 to close to 1. The interpretation values for *r* common in published literature and in the state of the art are: 0.10 ≤ *r* < 0.3 (small effect), 0.30 ≤ *r* < 0.5 (moderate effect) and *r* ≥ 0.5 (large effect) [17].

In parametric tests, the effect size measure was estimated using Cohen’s d. For the independent samples *t*-test, Cohen’s d was calculated as the difference between means divided by the estimated standardized deviation. The effect size paired samples *t*-test was based on the standard deviation of the differences or independent samples *t*-test. Quantification of the effect size magnitude was performed using the thresholds defined by Cohen [18]. The magnitude was assessed using the thresholds provided in Cohen’s paper, i.e., |d| < 0.2 “negligible”, |d| < 0.5 “small”, |d| < 0.8 “medium”, otherwise “large”.

## 3. Results

### 3.1. Microbiome Samples

In order to compare the microbiome’s composition, 24 detected bacterial taxa were chosen, based on the highest number of counts in all fecal samples (Table 1).

At the beginning of the study (T_0_) and after 4 weeks (T_1_), the microbiome parameters of a total of *N* = 30 subjects were examined. The sample at the 8-week time point (T_2_) was *N* = 29, as the number in the gluten group at the 8-week time point was *n* = 14 due to COVID-19 quarantine of one of the patients. The results of the Shapiro–Wilk test performed showed that the distributions for all parameters deviated from normality (*p*_Shapiro-Wilk_ < 0.05).

### 3.2. Changes in Microbiome 

#### 3.2.1. Changes in Microbiome after 4 Weeks of Gluten-Free Diet

**Figure 3:**
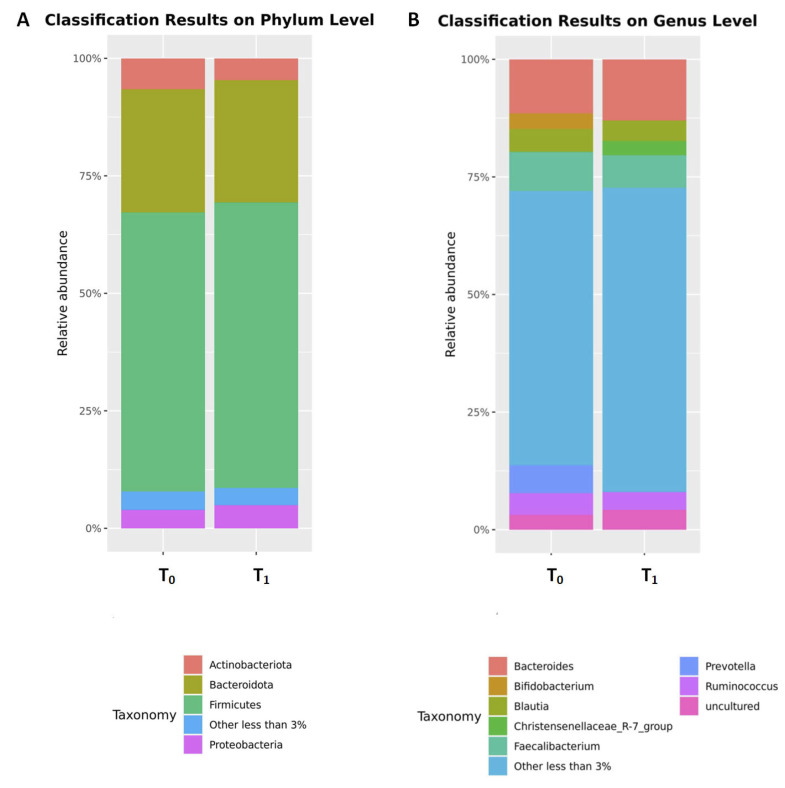
The comparison of the relative abundances of the top microbial phyla (**A**) and genera (**B**) before (T_0_) and after (T_1_) 4 weeks of a gluten-free diet. Only phyla and genera present at relative abundances > 3% are shown. Taxa with lower abundances are grouped as “other”.

After 4 weeks of a gluten-free diet, there was a significant increase in abundance of phyla *Desulfobacterota* and *Proteobacteria* and genera *Prevotella* and *Parasutterella*. In contrast, a significant decrease was observed for phylum *Actinobacteriota*, family *Coriobacteriaceae* and genus *Bifidobacterium* (Figure 3; Table 2). No significant changes in abundance were observed for the remaining bacterial taxa. The impact of the 4-week gluten-free diet on the microbiome is shown in Table 2 in detail.

#### 3.2.2. Differences between the Gluten and Placebo Groups in the Microbiome at the Time of Allocation (T_1_)

There were no significant differences in the abundance of 24 chosen bacterial taxa between the gluten and placebo groups at the time of allocation (T_1_), i.e., after 4 weeks on a gluten-free diet and before the start of taking capsules with gluten or rice starch (*p* > 0.05).

The greatest differences between groups (*r* ≥ 0.2) were found in the phylum *Cyanobacteria* and the family *Clostridiaceae* (greater means in the gluten group in both cases). No or almost no differences (*r* ≤ 0.03) were found between groups in phylum *Desulfobacterota*, families *Enterobacteriaceae* and *Victivallaceae*, and genera *Parasutterella* and *Slackia*. The results of the estimation of the differences between the gluten and placebo groups in the microbiome after 4 weeks on a gluten-free diet are shown in Table S1.

#### 3.2.3. Changes in Microbiome within the Gluten and Placebo Groups after 4 Weeks of Capsule Intake

We checked if there was any significant change in microbiome among patients enrolled in the same group (gluten or placebo) after 4 weeks of taking capsules when compared to the moment of allocation to the group, i.e., between time points T_1_ and T_2_.

A significant effect was observed only in the placebo group in phyla *Actinobacteriota* and *Bacteroidota* (increased abundance). Although no significant changes were observed in the gluten group (*p* < 0.05), changes at the trend level (0.05 ≤ *p* < 0.10) were observed in the family *Veillonellaceae* (decreased abundance). The effects of taking gluten capsules for 4 weeks in the gluten group and rice starch capsules for 4 weeks in the placebo group, along with a gluten-free diet, are shown in Table 3.

#### 3.2.4. Differences in Microbiome between the Gluten and Placebo Groups after Whole Intervention

There were no significant differences in the bacterial abundance within 24 chosen bacterial taxa between the gluten and placebo groups at the end of the intervention, i.e., at time point T_2_, after 8 weeks of gluten-free diet and 4 weeks of gluten/placebo intake (*p* > 0.05).

The greatest differences between the gluten and placebo groups (*r* = 0.28) were found for phyla *Bacteroidota* (greater means in the placebo group) and *Cyanobacteria* (greater means in the gluten group).

No or almost no differences (*r* ≤ 0.03) were found between the gluten and placebo groups for phyla *Actinobacteriota* and *Fusobacteriota*, family *Clostridiaceae*, genera *Dialister* and *Slackia*, and species *Akkermansia* (uncultured bacterium).

The results of the estimation of the differences between the gluten and placebo groups in the microbiome after 8 weeks on a gluten-free diet and 4 weeks of gluten/placebo intake are shown in Table S2.

### 3.3. Blood Parameter Analysis

The blood parameters were studied at three time points: baseline (T_0_), after 4 weeks (T_1_) and after 8 weeks (T_2_). The results of the performed Shapiro–Wilk test showed the normality of the distributions of the parameters TSH, FT4, FT3, anti-TPO, anti-TG and glucose (*p*_Shapiro-Wilk_ ≥ 0.05) and a deviation from normality for ESR and CRP (*p*_Shapiro-Wilk_ < 0.05).

At the beginning of the study (T_0_) and after 4 weeks (T_1_), the blood samples of a total of *N* = 30 subjects were examined. The sample at the 8-week time point (T_2_) was *N* = 28, as the number in the gluten group at the 8-week time point was *n* = 13. Also, in T_2_, in the placebo group, *n* = 14 for the ESR parameter because of a laboratory mistake in the case of one patient.

### 3.4. Changes in Blood Parameters

#### 3.4.1. Changes in Blood Parameters after 4 Weeks of a Gluten-Free Diet

There were no significant changes in blood parameters after 4 weeks of a gluten-free diet. The greatest changes were observed for anti-TPO and anti-TG antibody levels, where decreases in concentrations were observed with a small effect size. The smallest changes were observed for TSH levels with an effect close to 0. The results of the effects of the 4-week gluten-free diet on the blood parameters of the entire sample are shown in Table 4.

#### 3.4.2. Differences between the Gluten and Placebo Groups in the Blood Parameters at the Time of Allocation

Blood parameters differed significantly between the gluten and placebo groups at the time of allocation, i.e., in time point T_1_, after 4 weeks on a gluten-free diet and before start of taking capsules with gluten or rice starch only in case of glucose, with a large effect size. The gluten group had significantly lower glucose levels than the placebo group. 

In addition, differences at the trend level (0.05 ≤ *p* < 0.10) were evident for anti-TPO antibodies, for which lower concentrations were also observed in the gluten group. No differences in concentrations between the groups were observed for other blood parameters. The results of the estimation of the differences between the gluten and placebo groups in the blood parameters after 4 weeks of a gluten-free diet are shown in Table S3.

#### 3.4.3. Changes in Blood Parameters within the Gluten and Placebo Groups after 4 Weeks of Capsule Intake

We checked if there was any significant change in blood parameters among patients enrolled in the same group (gluten or placebo) after 4 weeks of taking capsules when compared to the moment of allocation to the group, i.e., between T_1_ and T_2_.

The only significant difference was observed in the placebo group for the anti-TPO antibody level. A gluten-free diet during weeks 5–8 with placebo capsule intake resulted in a significant decrease in anti-TPO antibody levels with a large effect size. In the group taking gluten, a decrease in the anti-TPO antibody levels to a trend level with a moderate effect size was also observed. The effects of taking gluten capsules for 4 weeks in the gluten group and rice starch capsules for 4 weeks in the placebo group, along with a gluten-free diet, are shown in Table 5.

#### 3.4.4. Differences in Blood Parameters between the Gluten and Placebo Groups after Whole Intervention

The blood parameters after an 8-week gluten-free diet and after a 4-week intake of the capsules differed significantly only in the case of the glucose levels; significantly higher glucose concentrations were found in the placebo group. In comparison after 4 weeks (time point T_1_) the effect size between the groups increased by 0.16, mainly due to a decrease in the standard deviation in both groups.

Trend-level differences were also observed between groups for the anti-TPO antibodies; higher concentrations were observed in the placebo group. Similarly, compared with the 4-week time point, there was a 0.08 increase in effect size between groups, mainly due to a decrease in standard deviation in the gluten group. The results of the estimation of the differences between the gluten and placebo groups in the blood parameters after whole intervention are shown in Table S4.

## 4. Discussion

The gut microbiome has been extensively researched due to its numerous associations with human health. Diet has a direct impact on the intestinal microbiome and, consequently, on health. The composition of the microbiome depends on the i.a. fiber intake in the form of vegetables, fruit and whole grains, as well as the amount of simple sugars and the type and amount of fat in the diet. 

Only women of normal weight were enrolled in the current study because it is known that obesity is also linked to altered microbiome [19,20,21].

There are more and more reports on the relationship between the microbiome and the human immune system and its potential impact on the development of autoimmune diseases. It has been demonstrated that AIT patients have altered gut microbiota in comparison to healthy controls [6].

However, the results of research on particular microbial alterations in patients with AIT are inconclusive. For instance, in some studies, the Bacteroidetes phylum or the Bacteroides genus were more abundant in AIT patients than in healthy controls [22,23], whereas in others, the opposite results were obtained [24]. Similarly, in the case of the *Firmicutes* phylum and the *Prevotella* species, opposite trends have been demonstrated [22,24].

On a taxonomic level, we identified seven bacteria that changed significantly in abundance during 4 weeks of a gluten-free diet: *Desulfobacterota*, *Proteobacteria*, *Prevotella* and *Parasutterella* increased, whereas *Actinobacteriota*, *Coriobacteriaceae* and *Bifidobacterium* decreased.

Our intervention caused enrichment in the *Desulfobacterota* phylum. It was previously shown to act in a pro-inflammatory manner and to have a detrimental impact on the intestines. *Desulfobacterota* also affects the nervous system and increased in the mouse model of Parkinson’s disease [25]. *Desulfobacterota* was also increased in diabetic retinopathy, and scientists suggested its role in disrupting energy metabolism [26]. Therefore, based on previous research, we can presume that its increase may be a sign of unfavorable changes in the microbiome. However, its role, particularly in AIT, has not been examined yet.

Another increased phylum after 4 weeks of the gluten-free diet was *Proteobacteria*. Its expansion has been associated with dysbiosis and a potentially higher risk of various diseases, especially metabolic and inflammatory disorders. For instance, an increase in *Proteobacteria* was related to the emergence of cardiovascular events in the general population [27], as well as to atherosclerotic plaque activation, potentially through pro-inflammatory effects [28]. It has been suggested that *Proteobacteria* plays an important role in inflammatory bowel diseases (IBD), mainly Crohn’s disease and ulcerative colitis [29,30].

*Proteobacteria* has not been extensively researched in thyroid diseases, especially in AIT. However, its increase has been demonstrated in thyroid carcinoma and Graves’ disease [31,32].

Shin et al. suggested that *Proteobacteria* is sensitive to any changes in the environment, such as the diet, and responds noticeably to them. Concurrently, it can be a sign of disease and microbial imbalance. Therefore, they propose time-series monitoring (such as the one in this study) as a better method of determining changes in *Proteobacteria* abundance than cross-sectional studies [33].

The unfavorable changes in microbiota could potentially be a result of the worse quality of patients’ diet, higher fat content and lower amount of fiber. It is a common problem in a gluten-free diet when based on highly processed packaged products. However, in this study, patients were educated about gluten-containing products and proper meal composition. They also received exemplary diet plans created by nutrition specialists, so low diet quality should not be an issue. In the case of a well-balanced, low-processed, and gluten-free diet, meals can be even more nutritious than in a regular diet containing gluten.

*Prevotella* also increased after 4 weeks of a gluten-free diet. *Prevotella* was found to flourish with non-Western diets rich in fiber [34]. It is presumed to contribute to weight loss, cholesterol decrease and glucose metabolism improvement [35,36,37]. However, in another study, *Prevotella copri* was correlated with insulin resistance [38]. Furthermore, *Prevotella* is associated with many other health conditions, including inflammatory autoimmune diseases like rheumatoid arthritis [39,40], local and systemic human infections, especially in the oral cavity or bacterial vaginosis, and HIV [41,42,43]. Therefore, *Prevotella* enrichment in this study can be a sign of increasing inflammation as well as of improvement in diet quality.

*Parasutterella*, another increased genus in this study, is known to be a core element of the human gut microbiome. One of the species from this genus, *P. excrementihominis*, has been correlated with inflammatory bowel disease, obesity, diabetes and fatty liver disease. Chen et al. demonstrated that *Parasutterella* may be associated with the onset and development of irritable bowel syndrome (IBS) and may contribute to chronic gut inflammation in IBS patients [44]. Furthermore, according to current studies, *Parasutterella* may play a crucial role in type 2 diabetes and obesity development [45]. A positive correlation of *Parasutterella* increase with the intake of carbohydrates was observed. This is consistent with the finding that a high-fat diet (HFD) results in diminished *Parasutterella* abundance both in animals and in human studies [46]. Besides growing inflammation, the increase in dietary carbohydrates and the decrease in fat may explain the changes in the current study.

In our research, a significant decrease after 4 weeks of the gluten-free diet was observed for the *Actinobacteriota* phylum (or Actinomycetota, previously called Actinobacteria) and the *Bifidobacterium* genus, which belongs to that phylum. It was demonstrated previously that *Actinobacteriota*, including 15 species of *Bifidobacterium*, diminished during human life [47]. 

*Actinobacteriota* and especially *Bifidobacterium* were also found to be decreased in Alzheimer’s disease [48]. In contrast, Liu et al. demonstrated a significant increase in the abundance of *Actinobacteriota* in the gut microbiome of patients with ankylosing spondylitis, which is a chronic inflammatory disease. Interestingly, ankylosing spondylitis patients also had a decreased relative abundance of the *Bifidobacterium* genus [49].

The beneficial impact of *Bifidobacterium* on human health is thoroughly supported by evidence [50,51]. Certain species of *Bifidobacterium* have been linked to anti-inflammatory effects and reduced intestinal permeability [52]. Moreover, supplementation with *Bifidobacterium* may result in the reduction of gut lipopolysaccharide levels and the improvement of the mucosal barrier in mice intestines [53,54]. However, the role of *Bifidobacterium* in autoimmune diseases, such as AIT, is still unclear. Some *Bifidobacterium* strains seem to be protective, whereas others may stimulate immunopathogenesis. It was observed in vitro that there were significant differences in levels of stimulated cytokine production and thus in promoted Th responses [55]. Xu et al. demonstrated that the relative abundance of the *Bifidobacterium* genus is positively correlated with a risk of type 1 diabetes and celiac disease [56].

*Coriobacteriaceae* is a bacterial family that belongs to the *Actinobacteriota* phylum and was also found to be diminished after 4 weeks of the gluten-free diet. This bacterial family is known to be crucial for host metabolic processes. However, it may also be considered as pathobiont because it has been associated with disorders such as bacteremia, periodontitis and vaginosis. *Coriobacteriaceae* was found to be positively associated with longevity, presumably due to a decrease in the risk of cardiovascular diseases [57]. It also has a protective effect on allergic rhinitis, which is a non-infectious inflammatory disease of the nasal mucosa [58]. *Coriobacteriaceae* abundance was diminished in patients with Crohn’s disease, compared to healthy controls and in metabolically unhealthy overweight/obese subjects, compared to metabolically healthy overweight/obese subjects [59,60]. On the other hand, *Coriobacteriaceae* was reduced in response to whole grain consumption, which was correlated with a significant decrease in the levels of plasma IL-6, an inflammatory marker [61].

Comparison of microbiome composition at time points T_1_ and T_2_ within the groups revealed a significant increase in the phyla *Actinobacteriota* and *Bacteroidota* in the placebo group. The placebo group took starch capsules during weeks 5–8, so they followed the gluten-free diet for the full 8 weeks. After the first 4 weeks, *Actinobacteriota* was decreased among all participants. The reason for the opposite effect in the second part of the gluten-free diet in the placebo group may be presumably higher fiber intake due to additional rice starch ingestion. *Actinobacteriota* and *Bacteroidota* were previously shown to be more abundant in the gut microbiota of Burkina Faso children compared with European children. The traditional rural African diet, followed by children in Burkina Faso, is rich in starch, fiber and plant polysaccharides and low in fat and animal protein [62].

No significant differences in microbiome were found between the gluten and placebo groups after the second part of the intervention when patients, apart from following a gluten-free diet, were also assigned to taking capsules with gluten or rice starch. The reason for the lack of significant differences could be the design of the intervention. It was based on the Di Sabatino study, where participants were given either 4.375 g/day gluten or rice starch (placebo) for 1 week [16]. However, due to practical reasons and patients’ adherence, we used only 2 g of gluten/day in capsules. Moreover, Di Sabatino explored individually perceived symptoms (intestinal and extraintestinal) and not the microbiome. Therefore, the amount of gluten used in our study could have been too small to induce significant alterations in the patients’ microbiome.

A diet containing 2 g of gluten per day was also examined by Hansen et al. as a low-gluten diet in comparison to a high-gluten diet containing 18 g of gluten per day. Healthy adults followed each diet regimen for 8 weeks separated by a washout period of at least six weeks with a habitual diet containing 12 g of gluten per day. The low-gluten diet resulted in a substantial reduction in 4 species of *Bifidobacterium*, which is consistent with the results of our 4-week gluten-free intervention. Moreover, a decrease in two species of *Dorea*, two species of the Lachnospiraceae family, *Blautia wexlerae*, *Anaeostipes hadrus* and *Eubacterium hallii* were observed. Concomitantly, unclassified species of Clostridiales and Lachnospiraceae increased during the low-gluten regimen compared with the high-gluten regimen [63].

Bonder et al. also studied changes in the microbiome during 4 weeks of a gluten-free diet but in healthy people. A decrease in *Veillonellaceae*, *Ruminococcus bromii* and *Roseburia faecis* and an increase in *Victivallaceae*, *Clostridiaceae*, ML615J-28, *Slackia* and *Coriobacteriaceae* were observed. They concluded that their intervention did not cause any major inflammatory or metabolic changes in intestinal function among healthy participants [64].

Significant, rather unfavorable changes in the gut microbiome of healthy people following a gluten-free diet for 1 month were found by De Palma et al. They observed a decrease in bacteria regarded as beneficial, such as *Bifidobacterium* and *Lactobacillus* and an enrichment in populations of opportunistic pathogens, such as *Escherichia coli* and total *Enterobacteriaceae* [65].

It has been proven that gut microbiota influences both immune system activity and thyroid function. AIT frequently coexists with celiac disease, which is attributed to a compromised intestinal barrier, leading to increased intestinal permeability [24]. Additionally, gut microbiota composition affects the availability of micronutrients essential for thyroid function [66]. Moreover, thyroid dysfunction may indirectly affect the concentrations of short-chain fatty acids (SCFA). SCFA are microbial metabolites that i.a. maintain intestinal homeostasis, modulate the immune response and have anti-inflammatory properties. In particular, butyrate seems important, as it has been associated with a reduction in proinflammatory factors and with an increased colonic population of Treg lymphocytes able to suppress auto-reactive immune responses [67]. Changes in SCFA concentrations were also shown as a result of a gluten-free diet. Zafeiropoulou et al. observed a decrease in butyric, propionic and valeric acids and an increase in acetic acid after 6 months of gluten elimination in celiac disease patients [68].

In the case of blood parameters, the only significant difference in our study was a decrease in anti-TPO levels in the placebo group between time points T_1_ and T_2_, so after 1 month of the gluten-free diet along with the rice starch capsule intake. This is partially consistent with the results obtained by Krysiak et al. In their study, the 6-month gluten-free diet reduced serum titers of anti-TPO and anti-Tg antibodies in euthyroid women with Hashimoto’s thyroiditis. Similarly, there were no significant differences in TSH and thyroid hormones FT4 and FT3 [9].

However, Pobłocki et al. acquired different results while examining the influence of a 12-month gluten-free diet on women with AIT. No differences were found between the control group and the gluten-free diet group in anti-TPO and anti-TG antibodies, FT3 or FT4 levels, but there was a significant reduction in TSH levels in the gluten-free diet group. Also, reduction of anti-TG concentrations in the gluten-free diet group was observed in statistical analyses performed separately for both groups [10]. Therefore, there is still not enough consistent data to explicitly assess the impact of a gluten-free diet on thyroid function in AIT.

This study had certain limitations. First, it was performed among women from the same area of residence (Pomeranian Voivodeship, Poland). Second, the sample size was relatively small due to financial and organizational constraints. Third, there was no control group composed of healthy people. It would also be best to compare microbiota and changes in blood parameters during a gluten-free diet among people without AIT. Lastly, the dietary regimen was not strictly controlled, so we cannot be certain about the gluten or fiber and macronutrients content in participants’ diet. Therefore, a large-scale study involving different populations, healthy controls and strict dietary supervision is needed to confirm our results.

## 5. Conclusions

Assessment of changes in the intestinal microbiome is limited due to complexity, numerous dependencies and variables regarding gut bacteria. However, deterioration of the microbial gut composition in AIT patients on a gluten-free diet is highly possible; therefore, there is an urgent need for more accurate research on a larger scale. Additionally, the benefits of a gluten-free diet in AIT are still questionable, so it should be implemented cautiously.

## Figures and Tables

**Table 1:** Bacterial taxa detected in patients’ fecal samples and chosen for further analysis.

Taxonomic Level	Bacterial Taxa
Phylum	*Actinobacteriota*, *Bacteroidota*, *Cyanobacteria*, *Desulfobacterota*, *Firmicutes*, *Fusobacteriota*, *Proteobacteria*, *Verrucomicrobiota*
Family	*Prevotellaceae*, *Veillonellaceae*, *Clostridiaceae*, *Coriobacteriaceae*, *Enterobacteriaceae*, *Victivallaceae*
Genus	*Bifidobacterium*, *Lactobacillus*, *Dialister*, *Prevotella*, *Alistipes*, *Escherichia-Shigella*, *Parasutterella*, *Slackia*
Species	*Escherichia coli*, *Akkermansia* (uncultured bacterium)

**Table 2:** Distribution of bacterial abundance at baseline (T_0_) and after 4 weeks of a gluten-free diet (T_1_) with test results within the entire sample, *N* = 30.

Bacteria	*n_pairs_*	Bacterial Abundance, Mdn (Q1–Q3)		*p*	*r*
		Baseline (T_0_)	After 4 Weeks (T_1_)		
Phylum					
*Actinobacteriota*	30	3199.0 (2165.0–5285.0)	2962.5 (1697.3–3626.8)	**0.038**	0.38
*Bacteroidota*	30	16,684.5 (11,372.5–18,349.8)	16,112.5 (14,660.0–18,170.0)	0.700	0.07
*Cyanobacteria*	30	3.0 (0–632.0)	58.5 (99.5–257.5)	0.639	0.07
*Desulfobacterota*	30	213.0 (155.0–333.0)	294.0 (159.8–454.8)	**0.021**	0.41
*Firmicutes*	30	34,854.5 (31,463.0–39,079.3)	37,647.5 (30,931.5–40,956.0)	0.221	0.23
*Fusobacteriota*	30	0 (0–0)	0 (0–0)	0.889	0.11
*Proteobacteria*	30	1667.0 (962.0–2440.0)	2408.5 (1402.0–4711.8)	**0.015**	0.44
*Verrucomicrobiota*	30	507.0 (194.5–1761.8)	734 (307.3–2109.8)	0.761	0.06
Family					
*Prevotellaceae*	30	1375.0 (17.5–4595)	1456.5 (2.0–3000.8)	0.105	0.30
*Veillonellaceae*	30	180.0 (8.5–1017.0)	95.0 (9.8–754.8)	0.146	0.22
*Clostridiaceae*	30	89.5 (38.8–245.0)	78.0 (15.8–565.8)	0.728	0.02
*Coriobacteriaceae*	30	967.5 (578.0–1992.5)	731.0 (288.3–1340.3)	**0.003**	0.52
*Enterobacteriaceae*	30	129.0 (44.0–337.5)	119.5 (23.0–518.8)	0.746	0.06
*Victivallaceae*	30	17.5 (0–38.8)	21.5 (0–48.5)	0.334	0.16
Genus					
*Bifidobacterium*	30	1238.5 (565.8–2983.0)	672.5 (309.3–1643.3)	**0.011**	0.46
*Lactobacillus*	30	4.5 (0–20.8)	2.5 (0–15.3)	0.158	0.30
*Dialister*	30	66.5 (0–999.0)	10.5 (0–667.0)	0.194	0.22
*Prevotella*	30	66.0 (9.3–3402.0)	93.0 (2.0–1610.3)	**0.044**	0.37
*Alistipes*	30	1249.5 (716.8–2385.5)	1332 (986.0–2608.0)	0.727	0.07
*Escherichia-Shigella*	30	78.0 (26.0–327.0)	99.5 (16.3–482.0)	0.729	0.07
*Parasutterella*	30	43.5 (14.3–153.8)	72.5 (18.3–266.3)	**0.008**	0.49
*Slackia*	30	20.5 (0–199.3 )	15.5 (0–182.8)	0.237	0.177
Species					
*Escherichia coli*	30	78.0 (26.0–327.0)	99.5 (16.3–482.0)	0.729	0.07
*Akkermansia*	30	409.5 (126.5–1256.0)	358.0 (207.5–1913.5)	1.000	0.02

In bold are marked statistically significant results, *p* < 0.05.

**Table 3:** Bacterial abundance between the 4-week gluten-free diet (T_1_) and 8-week gluten-free diet with 4-week intake of gluten/placebo capsules (T_2_) with test results within the groups.

Bacteria	*n_pairs_*	Group	Bacterial Abundance, Mdn (Q1–Q3)		*p*	*r*
			After 4 Weeks (T_1_)	After 8 Weeks (T_2_)		
Phylum						
*Actinobacteriota*	14	gluten	2622.5 (1964.2–3224.0)	2736.0 (1767.5–4918.2)	0.502	0.19
	15	placebo	2728.0 (1228.5–3531.5)	3412.0 (1565.0–4603.0)	**0.030**	0.56
*Bacteroidota*	14	gluten	15,816.5 (14,591.0–18,856.5)	16,310.0 (11,928.0–19,632.5)	0.542	0.18
	15	placebo	16,188.0 (15,573.0–17,939.0)	19,942.0 (15,962.0–21,380.0)	**0.015**	0.62
*Cyanobacteria*	14	gluten	186.5 (1.5–654.0)	111.5 (313.6–523.0)	1.000	0.01
	15	placebo	4.0 (0–151.5 )	0 (0–144.0)	0.906	0.16
*Desulfobacterota*	14	gluten	281.5 (183.5–496.5)	346.0 (187.5–517.2)	0.889	0.05
	15	placebo	335.0 (155.5–383.5)	266.0 (157.0–369.5)	0.303	0.28
*Firmicutes*	14	gluten	37,266.5 (33,142.2–40,886.0)	35,924.0 (30,814.8–41,329.8)	0.583	0.16
	15	placebo	35,393.0 (30,649.0–40,745.5)	35,828.0 (33,275.0–42,841.5)	0.188	0.35
*Fusobacteriota*	14	gluten	0 (0–0)	0 (0–0)	0.584	0.04
	15	placebo	0 (0–2.5)	0 (0–0)	0.178	0.36
*Proteobacteria*	14	gluten	3002.0 (1416.2–4748.5)	3488.5 (2149.5–5852.5)	0.326	0.28
	15	placebo	2462.0 (1749.5–4070.5)	2496.0 (1655.0–4494.5)	0.599	0.15
*Verrucomicrobiota*	14	gluten	500.5 (274.0–1210.0)	471.0 (171.0–2791.5)	0.235	0.34
	15	placebo	1273.0 (446.5–2138.0)	798.0 (356.0–1717.5)	0.421	0.22
Family						
*Prevotellaceae*	14	gluten	2349.5 (99.0–3476.2)	1682.0 (124.2–3387.2)	0.889	0.07
	15	placebo	745.0 (2.0–2363.0)	851.0 (0.0–4747.5)	0.255	0.22
*Veillonellaceae*	14	gluten	158.0 (9.8–961.0)	30.0 (0.0–480.0)	0.056	0.55
	15	placebo	74.0 (17.0–390.0)	109.0 (9.0–671.0)	0.889	0.04
*Clostridiaceae*	14	gluten	162.0 (35.0–1361.2)	192.0 (108.0–457.0)	0.328	0.25
	15	placebo	68.0 (11.0–459.5)	202 (35.5–695.5)	0.532	0.17
*Coriobacteriaceae*	14	gluten	1010.5 (415.5–1507.5)	1726.5 (8326.8–2225.0)	0.241	0.33
	15	placebo	654.0 (300.0–1169.5)	994.0 (454.5–1928.0)	0.117	0.42
*Enterobacteriaceae*	14	gluten	109.0 (19.2–648.2)	503.0 (54.2–2269.5)	0.780	0.08
	15	placebo	132.0 (35.0–317.0)	156.0 (47.5–427.0)	0.244	0.31
*Victivallaceae*	14	gluten	23.5 (0–48.5)	16.5 (0–67.2)	0.906	0.02
	15	placebo	21.0 (0–65.0)	20.0 (0–98.5)	0.359	0.14
*Genus*						
*Bifidobacterium*	14	gluten	731.0 (400.2–1523.2)	882.5 (560.2–1899.8)	0.358	0.26
	15	placebo	1154.7 (229.5–1292.0)	801.0 (470.5–1623.0)	0.184	0.33
*Lactobacillus*	14	gluten	5.0 (0–41.5)	1.5 (0–23.0)	1.000	0.06
	15	placebo	2.0 (0–8.5)	9.0 (0–20.5)	0.221	0.34
*Dialister*	14	gluten	7.5 (0–946.2)	0 (0–480.8)	0.236	0.311
	15	placebo	52.0 (0–390.0)	0 (0–660.5)	0.722	0.19
*Prevotella*	14	gluten	149.5 (6.5–1783.2)	100.5 (6.2–1946.5)	0.780	0.10
	15	placebo	6.0 (1.0–1585.5)	25.0 (0.0–3935.0)	0.197	0.31
*Alistipes*	14	gluten	1302.8 (990.2–2243.0)	1836.5 (904.8–2040.2)	0.217	0.34
	15	placebo	1207.0 (892.5–2973.0)	1125.0 (732.5–2637.0)	0.679	0.12
*Escherichia-Shigella*	14	gluten	41.0 (3.8–641.0)	489.0 (40.8–2268.8)	0.784	0.10
	15	placebo	132.0 (35.0–317.0)	156.0 (21.5–392.5)	0.244	0.31
*Parasutterella*	14	gluten	91.5 (22.2–334.0)	117.5 (34.8–280.5)	0.660	0.13
	15	placebo	80.0 (30.0–203.5)	68.0 (18.5–230.5)	0.315	0.30
*Slackia*	14	gluten	21.5 (0–498.8)	25.5 (63.1–229.8)	0.326	0.24
	15	placebo	16.0 (47.7–160.5)	9.0 (53.1–242.0)	0.760	0.02
Species						
*Escherichia coli*	14	gluten	41.0 (3.8–641.0)	489.0 (40.8–2094.8)	0.724	0.12
	15	placebo	132.0 (35.0–317.0)	156.0 (21.5–392.%)	0.244	0.31
*Akkermansia*	14	gluten	318.0 (272.8–1157.0)	338.0 (50.2–2727.0)	0.367	0.24
	15	placebo	852.0 (176.0–2105.5)	488.0 (711.1–1608.5)	0.530	0.18

In bold are marked statistically significant results, *p* < 0.05.

**Table 4:** Distribution of blood parameters at baseline (T_0_) and after 4 weeks of gluten-free diet (T_1_) with test results within the entire sample, *N* = 30.

Parameter	*n_pairs_*	Distribution of Blood Parameters, M (SD) ^1^		*p*	*d* ^2^
		Baseline (T_0_)	After 4 Weeks (T_1_)		
TSH [µIU/mL]	30	2.15 (1.2)	2.14 (1.08)	0.969	0.01
FT4 [ng/dL]	30	1.32 (0.20)	1.31 (0.20)	0.551	0.11
FT3 [pg/mL]	30	2.88 (0.32)	2.86 (0.33)	0.675	0.08
Anti-TPO [IU/mL]	30	152.84 (144.00)	145.31 (138.3)	0.154	0.27
Anti-TG [IU/mL]	30	244.39 (187.8)	218.99 (170.92)	0.142	0.28
Glucose [mg/dL]	30	87.47 (6.28)	88.07 (6.92)	0.546	–0.11
ESR [mm/h]	30	3.0 (2.0–8.0)	3.0 (2.0–8.0)	0.680	0.08
CRP [mg/L]	30	0.50 (0.50–0.78)	0.50 (0.50–1.10)	0.407	0.15

TSH, thyroid-stimulating hormone; FT4, thyroxine; FT3, triiodothyronine; Anti-TPO, thyroid peroxidase antibodies; Anti-TG, thyroglobulin antibodies; ESR, erythrocyte sedimentation rate; CRP, C-reactive protein. ^1^ For ESR, CRP the Mdn (Q1–Q3) was reported; ^2^ For ESR, CRP the r effect size was reported.

**Table 5:** Distribution of blood parameters between the 4-week gluten-free diet (T_1_) and 8-week gluten-free diet with 4-week intake of gluten/placebo capsules (T_2_) with test results within the groups.

Parameter	Group	Distribution of Blood Parameters, M (SD) ^1^		*p*	*d* ^2^
		After 4 Weeks (T_1_)	After 8 Weeks (T_2_)		
TSH [µIU/mL]	gluten	2.08 (0.87)	2.08 (0.78)	0.685	0.12
	placebo	2.20 (1.27)	2.06 (1.11)	0.626	0.13
FT4 [ng/dL]	gluten	1.27 (0.14)	1.32 (0.19)	0.175	−0.40
	placebo	1.34 (0.25)	1.34 (0.20)	0.978	−0.01
FT3 [pg/mL]	gluten	2.81 (0.34)	2.79 (0.34)	0.769	0.08
	placebo	2.90 (0.33)	2.89 (0.30)	0.882	0.04
Anti-TPO [IU/mL]	gluten	99.90 (88.0)	86.17 (69.98)	0.067	0.56
	placebo	190.71 (165.70)	184.56 (166.99)	**0.024**	0.66
Anti-TG [IU/mL]	gluten	215.05 (187.42)	236.42 (199.06)	0.722	0.10
	placebo	222.93 (159.23)	203.81 (153.56)	0.110	0.44
Glucose [mg/dL]	gluten	85.13 (6.16)	85.62 (4.96)	0.561	−0.17
	placebo	91.00 (6.55)	90.67 (4.40)	0.711	0.10
ESR [mm/h]	gluten	3.00 (2.50–8.00)	3.00 (3.00–9.00)	0.605	0.23
	placebo	3.00 (2.00–7.70)	3.00 (2.00–5.50)	0.313	0.20
CRP [mg/L]	gluten	0.60 (0.50–1.50)	0.50 (0.50–0.90)	0.121	0.42
	placebo	0.50 (0.50–0.70)	0.50 (0.50–0.55)	0.353	0.16

^1^ For ESR, CRP the Mdn (Q1–Q3) was reported; ^2^ For ESR, CRP the r effect size was reported. In bold are marked statistically significant results, *p* < 0.05.

## Data Availability

The data presented in this study are available on request from the author. The data are not publicly available due to ethical restrictions.

## Supplementary Materials

The following supporting information can be downloaded at: https://www.mdpi.com/article/10.3390/nu16050685/s1. Table S1: Bacterial abundance after 4 weeks of a gluten-free diet (T_1_) with test results between the gluten and the placebo groups; Table S2: Bacterial abundance after 8 weeks of a gluten-free diet with 4 weeks of gluten/placebo intake (T_2_) with test results between groups, *N* = 29; Table S3: Distribution of blood parameters after 4 weeks of a gluten-free diet (T_1_) with test results between the gluten and placebo groups; Table S4: Distribution of blood parameters after 8 weeks of a gluten-free diet with 4 weeks of gluten/placebo intake (T_2_) with test results between groups.
