# Supplementary material for: Gluten-Free Diet Alters the Gut Microbiome in Women with Autoimmune Thyroiditis
Source: Nutrients. 2024 Feb 28;16(5):685. doi: 10.3390/nu16050685 (PMC10935097; doi:10.3390/nu16050685)
Supplement: Supplementary file 1 [file nutrients-16-00685-s001.zip › Table S1.pdf]

Table S1. Bacterial abundance after 4 weeks of gluten-free diet (T_1) with test results between the gluten and the placebo groups.

Bacteria	Bacterial abundance, *Mdn (Q1 – Q3)*		*p*	*r*
	**Gluten group (n = 15)**	**Placebo group (n = 15)**		
Phylum				
Actinobacteriota	2657.0 (2029.5 – 3616.5)	2728 (1228.5 – 3531.5)	0.389	0.16
Bacteroidota	15386.0 (13822.0 – 18674.0)	16148.5 (15573.0 – 17939.0)	0.775	0.06
Cyanobacteria	185.0 (0 – 637.0)	4.0 (0 – 151.5)	0.217	0.23
Desulfobacterota	229.0 (174.5 – 490.0)	335.0 (155.5 – 383.5)	0.967	0.01
Firmicutes	38702.0 (33183.5 – 41703.5)	35393.0 (30649.0 – 40745.5)	0.539	0.12
Fusobacteriota	0 (0 – 0)	0 (0 – 2.5)	0.695	0.08
Proteobacteria	3366.9 (1296.5 – 4634.0)	2639.2 (1749.5 – 4070.5)	0.852	0.04
Verrucomicrobiota	431.0 (249.5 – 1100.0)	1273.0 (446.5 – 2138.0)	0.889	0.07
Family				
Prevotellaceae	2341.0 (8.0 – 3384.5)	745.0 (2.0 – 2363.0)	0.632	0.09
Veillonellaceae	293.0 (10.5 – 916.5)	74.0 (17.0 – 390.0)	0.723	0.07
Clostridiaceae	113.0 (29.5 – 1184.5)	68.0 (11.0 – 459.5)	0.245	0.22
Coriobacteriaceae	908.0 (330.5 – 1435.0)	654.0 (300.0 – 1169.5)	0.468	0.14
Enterobacteriaceae	101.0 (23.5 – 605.5)	132.0 (35.0 – 317.0)	0.934	0.02
Victivallaceae	22.0 (0 – 42.0)	21.0 (0 – 65.0)	0.966	0.01
Genus				
Bifidobacterium	780.0 (416.5 – 1755.0)	373.0 (229.5 – 1292.0)	0.300	0.19
Lactobacillus	5.0 (0 – 33.0)	2.0 (0 – 8.5)	0.584	0.10
Dialister	9.0 (0 – 853.5)	52.0 (0 – 390.0)	0.622	0.09
Prevotella	115.0 (3.0 – 1639.5)	6.0 (1 – 1585.5)	0.754	0.06
Alistipes	1196.0 (1021.5 – 2189.0)	1515 (892.5 – 2973)	0.653	0.09
Escherichia-Shigella	49.0 (7.5 – 599.0)	132.0 (35.0 – 317.0)	0.561	0.11
Parasutterella	65.0 (17.5 – 312.0)	80.0 (30.0 – 203.5)	0.983	0.01
Slackia	4.0 (0 – 455.5)	16.0 (0 – 160.5)	1.000	0.00
Species				
Escherichia coli	49.0 (7.5 – 599.0)	132.0 (35.0 – 317.0)	0.561	0.11
Akkermansia	297.0 (249.5 – 994.0)	852.0 (176.0 – 2105.0)	0.319	0.19
