# Supplementary material for: Gluten-Free Diet Alters the Gut Microbiome in Women with Autoimmune Thyroiditis
Source: Nutrients. 2024 Feb 28;16(5):685. doi: 10.3390/nu16050685 (PMC10935097; doi:10.3390/nu16050685)
Supplement: Supplementary file 1 [file nutrients-16-00685-s001.zip › Table S2.pdf]

Table S2. Bacterial abundance after 8 weeks of gluten-free diet with 4 weeks of gluten/placebo intake (T_2) with test results between groups, $N = 29$.

Bacteria	Bacterial abundance, *Mdn (Q1 – Q3)*		*p*	*r*
	**Gluten group (*n* = 14)**	**Placebo group (*n* = 15)**		
Phylum				
Actinobacteriota	2763.0 (1767.5 – 4918.2)	3412.0 (1565.0 – 4603.0)	0.880	0.03
Bacteroidota	16310.0 (11928.0 – 19632.5)	19942.0 (15962.0 – 21380.0)	0.146	0.28
Cyanobacteria	111.5 (3.2 – 523.0)	0.0 (0.0 – 144.0)	0.141	0.28
Desulfobacterota	346.0 (197.5 – 517.2)	266.0 (157.0 – 369.5)	0.266	0.21
Firmicutes	35924 (30814.8 – 41329.8)	35828 (33276.0 – 42841.5)	0.621	0.10
Fusobacteriota	0 (0 – 0)	0 (0 – 0)	0.975	0.01
Proteobacteria	3488.5 (2149.5 – 5852.2)	2496.0 (1655.0 – 4494.5)	0.451	0.15
Verrucomicrobiota	30.0 (0 – 480.8)	109 (9.0 – 671.0)	0.430	0.15
Family				
Prevotellaceae	1682.0 (124.2 – 33787.2)	851.0 (0.0 – 4747.5)	0.861	0.04
Veillonellaceae	30.0 (0 – 480.8)	109.0 (9 – 671.0)	0.430	0.15
Clostridiaceae	192.0 (108.0 – 457.0)	202 (35.5 – 695.5)	0.930	0.02
Coriobacteriaceae	1726.5 (326.8 – 2225.0)	994.0 (454.5 – 1928.0)	0.533	0.12
Enterobacteriaceae	1522.3 (542 – 2269.5)	1499.1 (47.5 – 427.0)	0.348	0.18
Victivallaceae	16.5 (0 – 67.2)	20.0 (0 – 98.5)	0.686	0.08
Genus				
Bifidobacterium	882.5 (560.2 – 1899.8)	801.0 (470.5 – 1623.0)	0.711	0.07
Lactobacillus	280.4 (0 – 23.0)	125.3 (0 – 20.5)	0.766	0.06
Dialister	0 (0 – 480.8)	0 (0 – 660.5)	0.943	0.02
Prevotella	100.5 (6.2 – 1946.5)	25.0 (0 – 3935.0)	0.644	0.09
Alistipes	1836.5 (904.8 – 2040.2)	1125.0 (732.5 – 2637.0)	0.451	0.15
Escherichia-Shigella	489.0 (40.8 – 2268.8)	156.0 (21.5 – 392.5)	0.419	0.15
Parasutterella	117.5 (34.8 – 280.5)	68.0 (18.5 – 230.5)	0.499	0.13
Slackia	25.5 (0 – 229.8)	9.0 (0 – 242.0)	0.873	0.03
Species				
Escherichia coli	489.0 (40.8 – 2096.8)	156.0 (21.5 – 392.5)	0.445	0.15
Akkermansia	338.0 (50.2 – 2727.0)	488.0 (170.0 – 1608.5)	1.000	0
