# Supplementary material for: Gluten-Free Diet Alters the Gut Microbiome in Women with Autoimmune Thyroiditis
Source: Nutrients. 2024 Feb 28;16(5):685. doi: 10.3390/nu16050685 (PMC10935097; doi:10.3390/nu16050685)
Supplement: Supplementary file 1 [file nutrients-16-00685-s001.zip › Table S3.pdf]

Table S3. Distribution of blood parameters after 4 weeks of gluten-free diet (T_1) with test results between the gluten and placebo groups.

Parameter	Distribution of blood parameters, *M (SD)*[1]		*p*	*d*[2]
	**Gluten group (*n* = 15)**	**Placebo group (*n* = 15)**		
TSH [µIU/mL]	2.08 (0.87)	2.20 (1.27)	0.774	-0.11
FT4 [ng/dl]	1.27 (0.14)	1.34 (0.25)	0.337	-0.36
FT3 [pg/ml]	2.81 (0.34)	2.90 (0.33)	0.455	-0.28
Anti-TPO [IU/ml]	99.90 (88.0)	190.71 (165.7)	0.075	-0.69
Anti-TG [IU/ml]	215.05 (187.42)	222.93 (159.23)	0.902	-0.05
Glucose [mg/dl]	85.13 (6.16)	91.00 (6.55)	**0.018**	-0.92
ESR [mm/h]	3.0 (2.50 – 8.00)	3.00 (2.00 – 7.50)	0.640	0.09
CRP [mg/l]	0.60 (0.50 – 1.50)	0.50 (0.50 – 0.70)	0.206	0.24

TSH, thyroid-stimulating hormone; FT4, thyroxine; FT3, triiodothyronine; Anti-TPO, thyroid peroxidase antibodies; Anti-TG, thyroglobulin antibodies; ESR, erythrocyte sedimentation rate; CRP, C-reactive protein.

[1] For ESR, CRP the *Mdn* (*Q1 – Q3*) was reported;
[2] For ESR, CRP the *r* effect size was reported.
