# Supplementary material for: Gluten-Free Diet Alters the Gut Microbiome in Women with Autoimmune Thyroiditis
Source: Nutrients. 2024 Feb 28;16(5):685. doi: 10.3390/nu16050685 (PMC10935097; doi:10.3390/nu16050685)
Supplement: Supplementary file 1 [file nutrients-16-00685-s001.zip › Table S4.pdf]

Table S4. Distribution of blood parameters after 8 weeks of gluten-free diet with 4 weeks of gluten/placebo intake (T_2) with test results between groups

Parameter	Distribution of blood parameters, *M (SD)*[1]		*p*	*d*[2]
	**Gluten group (n = 13)**	**Placebo group (n = 15)**[3]		
TSH [μIU/mL]	2.08 (0.78)	2.08 (1.11)	0.956	0.02
FT4 [ng/dl]	1.32 (0.19)	1.34 (0.20)	0.784	-0.11
FT3 [pg/ml]	2.79 (0.34)	2.89 (0.30)	0.409	-0.32
Anti-TPO [IU/ml]	86.17 (69.98)	184.56 (166.99)	0.051	-0.77
Anti-TG [IU/ml]	236.42 (199.06)	203.81 (153.56)	0.636	0.18
Glucose [mg/dl]	85.62 (4.96)	90.67 (4.40)	**0.009**	-1.08
ESR [mm/h]	5.0 (3.00 – 9.00)	3.00 (2.00 – 5.50)	0.148	0.28
CRP [mg/l]	0.60 (0.50 – 0.90)	0.50 (0.50 – 0.55)	0.129	0.29

TSH, thyroid-stimulating hormone; FT4, thyroxine; FT3, triiodothyronine; Anti-TPO, thyroid peroxidase antibodies; Anti-TG, thyroglobulin antibodies; ESR, erythrocyte sedimentation rate; CRP, C-reactive protein.

[1] For ESR, CRP the *Mdn* (*Q1 – Q3*) was reported;
[2] For ESR, CRP the *r* effect size was reported.
[3] *n* = 14 for ESR
